# Lip and oral cavity cancer in Iran from 1990 to 2019 based on the global burden of disease study

**DOI:** 10.1038/s41598-025-92090-w

**Published:** 2025-03-03

**Authors:** Leyla Roghanizadeh, Saede Atarbashi-Moghadam, Fatemeh Masaebi, Alireza Akbarzadeh Baghban

**Affiliations:** 1 Iranian Center for Endodontic Research, Research Institute for Dental Sciences, Shahid Beheshti University of Medical Sciences, Tehran, 1983963113 Iran; 2 Department of Oral and Maxillofacial Pathology, School of Dentistry, Shahid Beheshti University of Medical Sciences, Tehran, Iran; 3 Social Determinants of Health Research Center, Health and Metabolic Diseases Research Institute, Zanjan University of Medical Sciences, Zanjan, Iran; 4 Social Determinants of Health Research Center, Health and Metabolic Diseases Research Institute, Zanjan University of Medical Sciences, Zanjan, Iran; 5 Proteomics Research Center, Department of Biostatistics, School of Allied Medical Sciences, Shahid Beheshti University of Medical Sciences, Tehran, 1971653313 Iran

**Keywords:** Cancer epidemiology, Global burden of disease, Joinpoint regression, Morbidity, Mortality, Oral cancers, Trend, Cancer, Health care

## Abstract

The global burden of disease (GBD) provides valid and reliable assessments of the burden of various diseases. The present study aimed to evaluate the trends of incidence, mortality, and morbidity of lips and oral cavity cancers (LOCC) in Iran from 1990 to 2019 according to GBD 2019 data. Indicators including prevalence, incidence, death, years of life lost (YLL), years lived with disability (YLD), disability adjusted life years (DALY), and mortality-to-incidence ratio (MIR) for two genders and three age groups including 15–49 years, 50–69 years, and 70 + years in Iran were extracted from GBD 2019/calculated. The annual percentage changes of different indicators were analyzed. A joinpoint regression analysis was used to examine the pattern of changes. LOCC incidence in Iran has increased during the studied period, in both genders and all age groups. The average annual percent change (AAPC) of incidence increase was much higher in women (1.36) than in men (0.007), while the highest growth was observed in middle-aged women from 2010 to 2015 (6.9). The general trend (AAPC (Confidence interval (CI)) of YLL in total patients (-0.20 (-0.23, -0.17)), and DALY in total patients (-0.17 (-0.21, -0.14)) had a significant decrease. However, YLL in women (0.48 (0.44, 0.50)) and DALY in women (0.51 (0.48, 0.54)) significantly increased. Moreover, MIR has been declining during the studied period except for the time range of 2002 to 2010, in each of the two sexes (-0.46 in men and − 0.64 in women) and both together (-0.53). A declining trend of YLL/DALY/MIR, and an improvement in screening/diagnosis/treatment/patient care of LOCC in Iran has happened that still needs to be improved. Due to the increase in incidence/mortality/morbidity of LOCC in Iranian women, risk factors exposure and disease screening should be followed seriously.

**Supplementary Information:**

The online version contains supplementary material available at 10.1038/s41598-025-92090-w.

## Introduction

Lips and oral cavity cancers (LOCC) are defined as “malignant neoplasms, stated or presumed to be primary, of specified sites, except of lymphoid, haematopoietic, central nervous system or related tissues” [International Statistical Classification of Diseases and Related Health Problems (ICD) -11, 2B60-2B6Z]^1^. The involved sites include tongue, floor of the mouth, lips, hard palate, oral mucous membrane, gingiva, and the retromolar area^2^. Microscopically, squamous cell carcinoma (SCC) accounts for more than 90% of malignant tumors of the oral cavity and oropharynx, and the malignant mesenchymal neoplasms must be excluded from LOCC^1^. The same as other parts of the upper respiratory/digestive tract, the use of tobacco and alcohol have a strong and synergistic association with occurrence of the abovementioned malignancies^1^, while lip cancers are intensely related to ultraviolet radiation from exposure to sunlight^2^.

A description of the epidemiological situation of LOCC in different areas/countries of the world is of particular importance because it can be a guide for policymakers in planning and implementing an effective prevention and control program^3^. The incidence rates of LOCC have been continuously increasing with different geographical distribution in the different regions of the world^4^. South Asian countries including India, Pakistan, Afghanistan, Bangladesh, Sri Lanka, Bhutan, Nepal, Iran, and Maldives have the highest increase rates and are especially affected by LOCC. In some of these countries, oral cancer is the first or second most common cancer^4,5^. Based on variables such as the aging population, increased life expectancy, and exposure to certain risk factors such as smoking, an increasing trend of oral cancer in Iran can be expected^6^. The 5-year survival rate in patients with Oral SCC in Iran was reported to be ~ 40–49% ^7^. Furthermore, a cost-of-illness study indicated that in 2014, the economic burden of oral cancer in Iran reached more than $31 million, most of which was related to the loss of productivity mainly due to premature death in men^8^.

The global burden of disease (GBD) study started 30 years ago to provide valid and reliable evaluations of outcomes of various diseases. Led by the “Institute for Health Metrics and Evaluation” (IHME), GBD has the most international collaborations with medical scientists and practitioners from more than 150 countries. GBD is a powerful source for evaluating alterations in human health in this century^9,10^. GBD 2019 reflected advanced performance and standardization, introduced different models for outcomes of nearly 370 diseases/injuries, and offered a thorough perspective on worldwide health^11,12^.

Indicators including the death, prevalence, age-standardized incidence rates (ASIRs), age-standardized mortality rate (ASMR), years of life lost (YLL), and years lived with disability (YLD) can demonstrate the different aspect of diagnosis and treatment of a disease in a specific geographic area. Moreover, disability adjusted life years (DALY) which is the sum of YLL and YLD may be considered as one of the most important indicators of patients’ care. Additionally, mortality-to-incidence ratio (MIR) is another useful indicator which can show the status of cancer screening, diagnosis, treatment, and the relevant high or low mortality in a geographic area^4,13,14^. Although, indices related to the quality of patient’s life are difficult to measure and are not included in GBD 2019^15^.

This study aimed to investigate the burden of LOCC in Iran for thirty years from 1990 to 2019. For this purpose, prevalence, ASIR, ASMR, YLL, YLD, and DALY indices of LOCC in Iran in the years under review were extracted from the GBD database and analyzed.

## Methods

This observational study was approved by ethics committee of Shahid Beheshti University of Medical Sciences (IR.SBMU.RETECH.REC.1402.178).

### Obtaining and estimation process of GBD cancer data

Since there may not be complete mortality or morbidity data for every age, sex, and year, to complete the GBD data, methods of estimation is also used in GBD^16^. In the case of GBD cancer, the original data is obtained from case registrations in each country, autopsies, and cancer registry databases. The estimation process of GBD cancer data begins with death/mortality rates. When the incidence of cancer is registered but the mortality is not recorded, the incidence data are used to model mortality by multiplying incidence with a separately modeled MIR. These estimates for mortality are added to available cancer mortality statistics and are used in a cause of death ensemble model (CODEm) estimation, which is necessary to have a thorough mortality rate because original mortality data of every cause of death/cancer are not available for every age, sex, location, and year estimated by GBD database^13,16^.

Among the indicators that measure morbidity, YLL measures the reduction in life expectancy due to LOCC and is calculated as the number of deaths × the standard life expectancy at age of death. YLD is another index that shows the attenuation of a person’s quality of life due to injury or illness. It is calculated as the number of new cases of a disease × a disability weight × the average time a person lives with the disease before remission or death. DALY, which is the sum of the above two indicators (DALY = YLL + YLD), shows the total morbidity and health loss resulting from fatal and non-fatal illnesses^17,18^.

### Search strategy and data extraction

The data on the burden of LOCC for Iran and the relevant indicators were extracted from the GBD 2019 database with the following address: http://ghdx.healthdata.org/gbd-results-tool^19^. A combination of summary metrics was used to quantify the burden of LOCC including deaths, ASIR, ASMR, prevalence, YLL, YLD, and DALY. For this purpose, “Cause of death or injury” and “Lip and oral cavity cancer” were selected in the first box and the “Cause” box, respectively. Rate was the selected unit. Then, the LOCC “Measures” of death, incidence, and prevalence for both sexes in Iran from 1990 to 2019 were searched.

According to GBD age categories and similar articles^13,18^, age-standardized and three age groups were adopted including 15–49 years, 50–69 years, and 70 + years.

### Statistical analysis

According to the requested indicators, the result files were received from GBD 2019 in Excel sheets and stored.

Decrease accuracy in the data obtained from the estimations in GBD can be attributed to variability of estimates resulting from sampling errors, and non-sampling errors related to data sources and modeling. Therefore, the uncertainty intervals (UI) for estimations were analyzed by Monte Carlo simulation^13^ with 1,000 draws taken for each studied cause/disease, sex, age, and year.

In the present study, joinpoint regression analysis was employed by using the Joinpoint Regression Software (Joinpoint Version 4.8.0.1, Surveillance Research Program, National Cancer Institute, USA) to examine the pattern of changes in the indicators of LOCC and calculate the annual percent changes (APCs) of different indices. Joinpoint regression is a statistical method used to identify and analyze changes in trends over time. It allows for the detection of join points, which represent points at which the slope of a trend changes significantly^20^. The joinpoint regression model assumes that the observed data can be represented by a series of connected line segments, with each segment having a distinct slope. The number and locations of join points in the data are estimated based on statistical criteria. The process of joinpoint regression involves the following steps: 1, Data preparation, the data should be organized in chronological order, typically with a dependent variable or outcome variable measured at different time points; 2, Join point detection, the algorithm searches for join points by fitting regression models with different numbers of join points and selecting the optimal model that minimizes the residual sum of squares; 3, Join point estimation, once the join points are detected, the model estimates the locations of the join points and the associated changing slopes. Finally, the statistical significance of each join point and its associated slope change were assessed through hypothesis testing^21,22^.

## Results

**Fig. 1:**
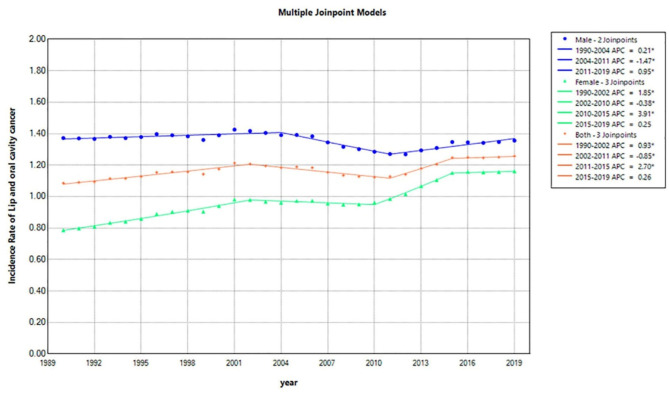
The multiple joinpoint regression model of the incidence rate of lip and oral cavity cancer in Iran between 1990 and 2019 for male, female, and both together based on the global burden of disease database. It can be observed that there are two join points for the incidence of abovementioned cancers in male, three in female, and three in all involved patients, respectively. APC, Annual percentage change.

**Fig. 2:**
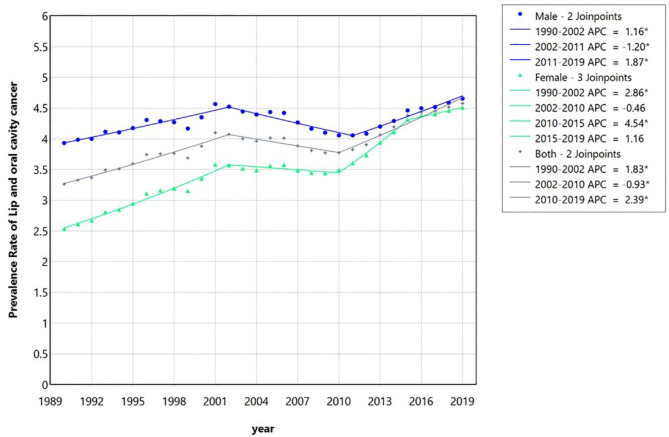
The multiple joinpoint regression model of the prevalence rate of lip and oral cavity cancer in Iran between 1990 and 2019 for male, female, and both together based on the global burden of disease database. As it can be observed, there are two join points for the prevalence of the aforementioned cancers in male and both genders patients; however, there are three join points in analysis of female patients. APC, Annual percentage change.

The descriptive statistics for the incidence, prevalence, and MIR of LOCC in 1990, 1995, 2000, 2005, 2010, 2015, and 2019 are shown in Table 1, separately in two genders and both. Moreover, the output of the analysis performed by the joinpoint software on the incidence, MIR, prevalence, YLD, YLL, and DALY data of LOCC in Iran during the study period are presented in the Supplementary files 1–4. The output of the joinpoint regression model for the incidence trend analysis can be observed in Table 2; Fig. 1. The joinpoint regression output for the prevalence is presented in Table 3; Fig. 2. For women who had 3 join points and 4 time periods, in two periods from 1990 to 2002 (the first period) and from 2010 to 2015 (the third period), significant increases in incidence and prevalence were seen. The APCs (95% Confidence interval (CI)) of incidence in women were 1.85 (1.68, 2.02) (1st period) and 3.91 (3.32, 4.78) (3rd period), respectively. The APCs of prevalence in women were 2.86 (2.57, 3.15) (1st period) and 4.54 (3.59, 6.63) (2nd period), respectively. For men, who had 2 join points and 3 time periods, there were significant increases in incidence between 1990 and 2004 and 2011 to 2019, with APCs of incidence equal to 0.21 (0.08, 0.36) and 0.95 (0.65, 1.33), respectively. Regarding the prevalence in men, significant increases occurred between 1990 and 2002 and 2011 to 2019, and APCs of prevalence were equal to 1.16 (0.88, 1.48) and 1.87 (1.33, 2.67), respectively. The rates of increase in both incidence and prevalence in women were much higher than in men.

The most obvious issues have been the upward trends of the both incidence and prevalence of LOCC in female patients in Iran from 1990 to 2019 with three join points. Furthermore, generally in the total 30-year time period, a significant increase in the LOCC incidence in both genders together was observed (Average APC (AAPC) = 0.52, 95% CI (0.44, 0.58). This total increase was related to female patients (with a significant AAPC = 1.36 (1.29, 1.42); while just a small increase in the incidence of LOCC in male patients had occurred (with AAPC = 0.007 (-0.05, 0.07).

**Fig. 3:**
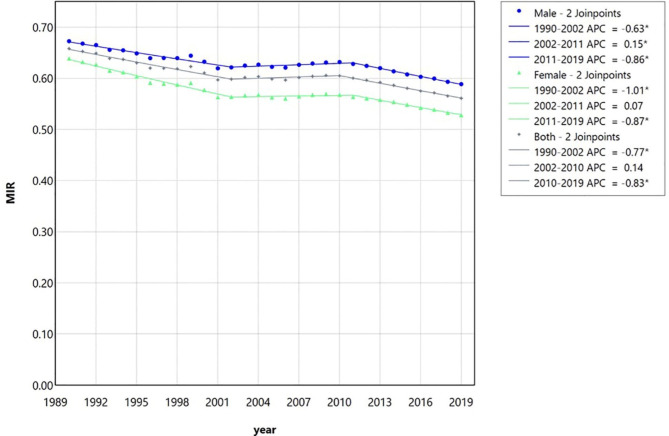
The multiple joinpoint regression model output for the trend analysis of the calculated mortality-to-incidence (MIR) ratio of lip and oral cavity cancer in Iran between 1990 and 2019 for male, female, and both together based on the global burden of disease database. Except for the middle period which the trend is somewhat upward, at the beginning and end of the period, as well as in general, there is a downward trend which shows the decreasing trend of mortality per incidence of the studied cancers. APC, Annual percentage change.

Table 4; Fig. 3 demonstrate the aforementioned regression model output for the calculated MIR as a multifactorial indicator. Except for the time range of 2002 to 2010, this index has been declining in the beginning and the end of the studied time period, in each of the two sexes and in both together.

**Fig. 4:**
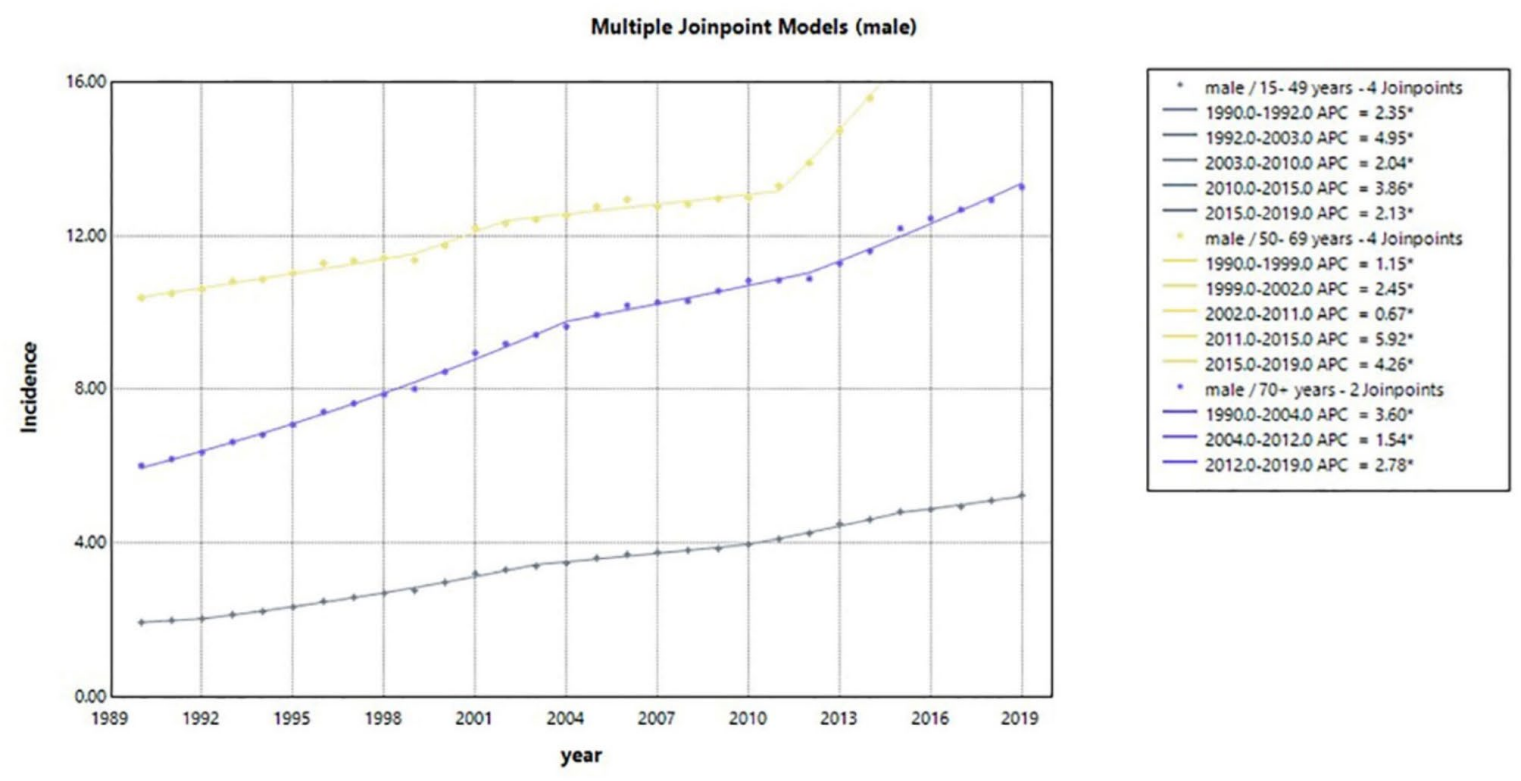
Temporal trends in LOCC estimated annual percentage change from 1990 to 2019 in male patients for three age groups (15–49 year, 50–69 year, and 70 + year). LOCC, lip and oral cavity cancers.

**Fig. 5:**
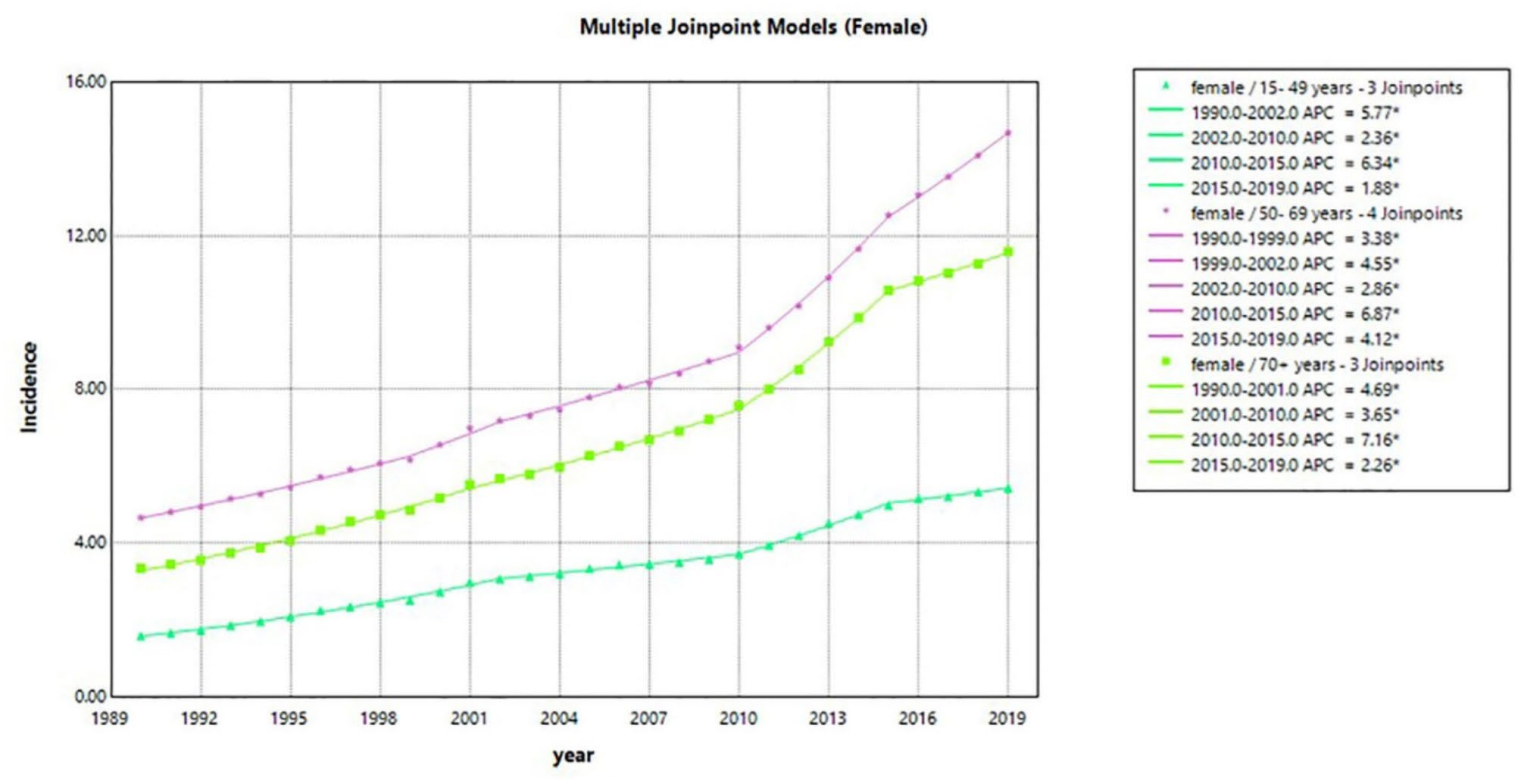
Temporal trends in LOCC estimated annual percentage change from 1990 to 2019 in female patients for three age groups (15–49 year, 50–69 year, and 70 + year). LOCC, lip and oral cavity cancers; APC, Annual percentage changes.

**Fig. 6:**
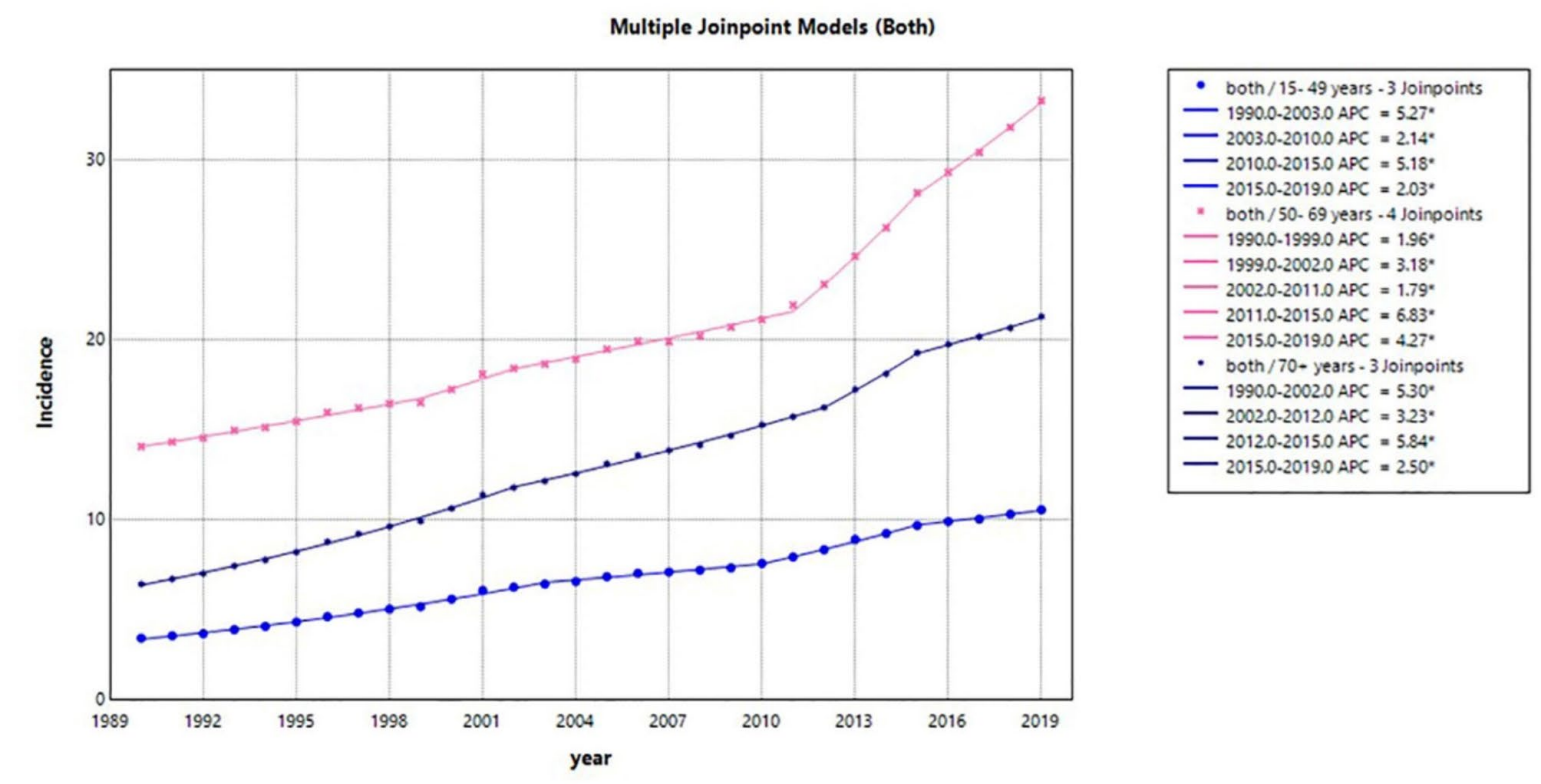
Temporal trends in LOCC estimated annual percentage change from 1990 to 2019 in patients of both genders for three age groups (15–49 year, 50–69 year, and 70 + year). LOCC, lip and oral cavity cancers; APC, Annual percentage changes.

Table 5; Figs. 4, 5 and 6 display the incidence of LOCC in three age groups. The results are presented separately for male and female patients, as well as for both genders combined. Upon initial inspection, two main conclusions can be drawn about the overall trend of incidence in LOCC. Firstly, the incidence exhibited an upward trend across all age and sex groups during the study period. Secondly, middle-aged populations of both genders experienced higher rates of incidence compared to other age groups. However, it should be noted that middle-aged women had a higher incidence rate than men.

Analyzing the incidence rates of 15-49-year-old population in both sexes together, we observed three join points in 2003, 2010, and 2015. These join points resulted in four distinct periods with different trends. Furthermore, the estimated APCs for incidence rates within specific time intervals were statistically significant, with values of 5.3, 2.1, 5.2, and 2.0 for the time intervals of 1990–2003, 2003–2010, 2010–2015, and 2015–2019, respectively. In the 15-49-year-old population, the most significant increase occurred in the first period (1990–2003) with an APC of 5.3 per 100,000. Similarly, in the middle-aged group, incidence rates had four join points in 1999, 2002, 2011, and 2015, leading to five distinct trends within the time intervals of 1990–1999, 1999–2002, 2002–2011, 2011–2015, and 2015–2019. The estimated APCs for these trends were 2.0, 3.2, 1.8, 6.8, and 4.3 in incidence rates, respectively. For the elderly population, three join points were identified in 2002, 2012, and 2015. This resulted in four distinct periods: 1990–2002, 2002–2012, 2012–2015, and 2015–2019. The related APCs were found to be 5.3, 3.2, 5.8, and 2.5, respectively. In the population of + 70 years, the most significant increase occurred from 2012 to 2015.

**Fig. 7:**
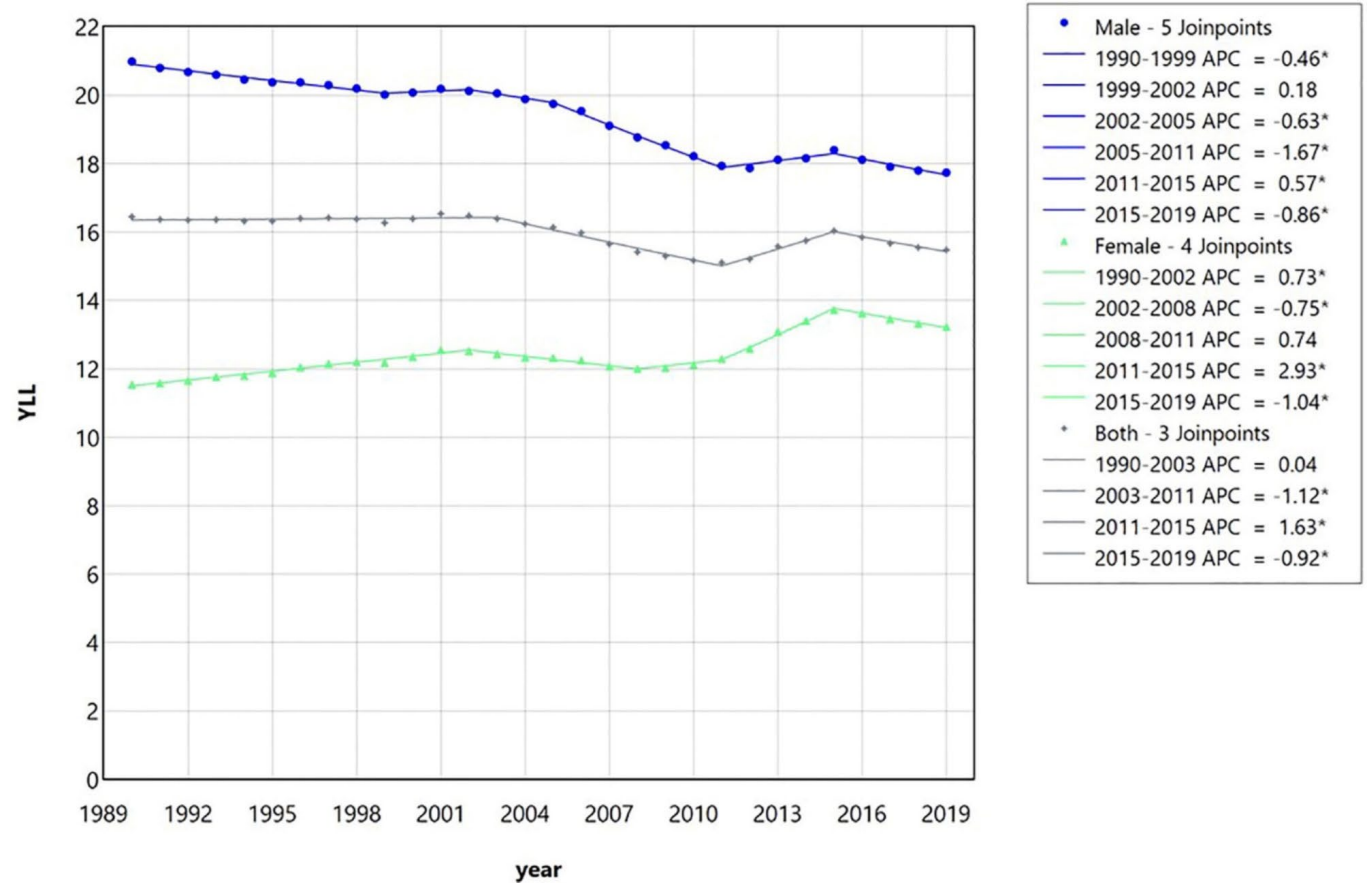
The trend analysis via multiple joinpoint regression model of the years of life lost (YLL) due to lip and oral cavity cancer in Iran between 1990 and 2019 for male, female, and both together based on the global burden of disease database. Despite the fluctuations of trends, generally, a decrease in men, an increase in women, and a low decrease in both sexes together can be observed. APC, Annual percentage change.

**Fig. 8:**
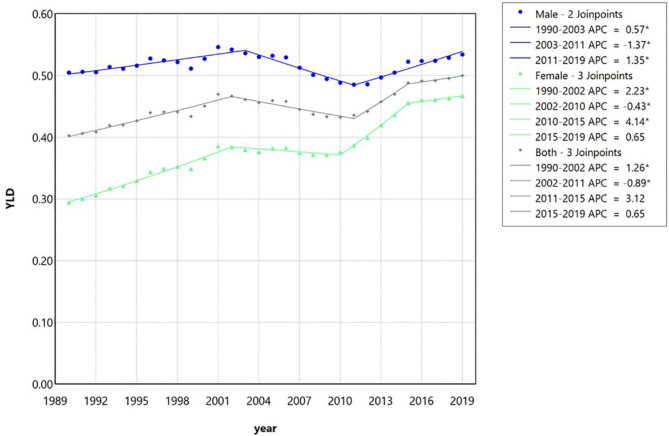
The trend analysis via multiple joinpoint regression model of the years lived with disability (YLD), due to lip and oral cavity cancer in Iran between 1990 and 2019 for male, female, and both together based on the global burden of disease database. Despite the small decrease in the middle years of the evaluated period, an increasing trend can be seen in male and female patients and all patients together. In women, there is the highest slope of YLD increase. APC, Annual percentage change.

**Fig. 9:**
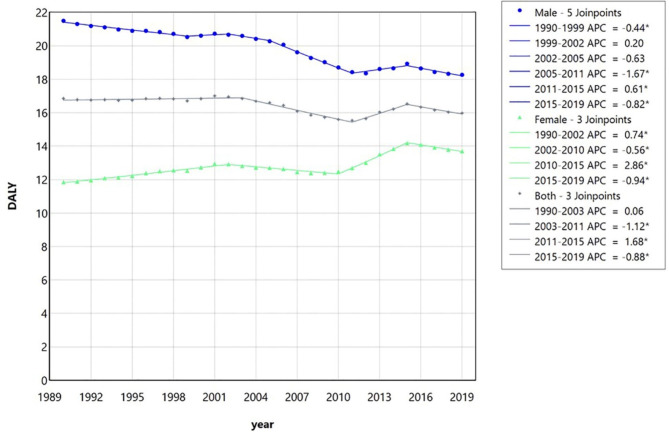
The trend analysis via multiple joinpoint regression model of the disability adjusted life years (DALY) due to lip and oral cavity cancer in Iran between 1990 and 2019 for male, female, and both together based on the global burden of disease database. In the DALY trend, despite the fluctuations, a decreasing trend can be seen in male patients and in all patients, and an increasing trend in female patients. APC, Annual percentage change.

Table 6 shows the indicators related to morbidity and patient care including YLL, YLD, and DALY per 100,000 (95% UI) of LOCC for males, females, and the entire population of Iran between 1990 and 2019. Moreover, the trend analyses through the joinpoint regression model for YLL, YLD, and DALY of the abovementioned cancers in Iran for the studied period are presented in Table 7; Figs. 7, 8 and 9, respectively. The most heterogeneity and join points were observed in the trend of years of life lost (YLL) (with five join points in males, four in females, and 3 in both sexes together) (Fig. 7). The general trend of YLL can be expressed as a significant decrease in men (AAPC=-0.58, 95% CI (-0.60, -0.56)); a significant increase in women (AAPC = 0.48 (0.44, 0.50)); and a significant decrease in the entire population (AAPC=-0.20 (-0.23, -0.17)).

Regarding the years lived with disability (YLD) (Fig. 8), there was an overall significant increase in each of the two sexes and in both in the reviewed period; the lowest increase in men and the highest increase in women were observed (significant AAPC of men = 0.24, 95% CI (0.15, 0.33); significant AAPC of women = 1.60 (1.50, 1.67); and significant AAPC of the entire population = 0.76 (0.64, 0.84)).

Disability adjusted life years (DALY) which one of its components is YLL, largely shows the pattern of YLL changes in the multiple joinpoint regression analysis (Fig. 9). The trend of DALY in men with 5 join points indicates a generally significant decrease, AAPC=-0.56, 95% CI (-0.58, -0.54). Although, DALY in the affected women with 3 join points reveals a general significant increasing trend, AAPC = 0.51 (0.48, 0.54). In addition, DALY of the total patients with the presence of 3 join points shows a decreasing trend, AAPC=-0.17 (-0.21, -0.14) which indicates that the effect of reducing DALY in men was greater than the effect of increasing it in women in this period.

**Table 1:** The incidence and prevalence rate per 100,000 (95% uncertainty intervals), and the mortality-to-incidence (MIR) ratio of lip and oral cavity cancer in Iran for the selected years from the global burden of disease database.

	Sex	1990	1995	2000	2005	2010	2015	2019
Incidence	Male	1.37 (1.07, 1.68)	1.38 (1.15, 1.62)	1.39 (1.23, 1.55)	1.39 (1.29, 1.49)	1.28 (1.20, 1.38)	1.35 (1.23, 1.45)	1.35 (1.21, 1.52)
	Female	0.79 (0.66, 0.90)	0.86 (0.74, 0.96)	0.94 (0.85, 1.03)	0.97 (0.89, 1.06)	0.96 (0.88, 1.04)	1.15 (1.05, 1.25)	1.16 (1.04, 1.30)
	Both	1.08 (0.88, 1.27)	1.13 (0.97, 1.28)	1.17 (1.07, 1.27)	1.19 (1.11, 1.26)	1.12 (1.05, 1.19)	1.25 (1.16, 1.33	1.26 (1.15, 1.38)
Prevalence	Male	3.93 (3.04, 4.93)	4.18 (3.43, 5.01)	4.35 (3.80, 4.99)	4.44 (4.02, 4.89)	4.06 (3.67, 4.48)	4.46 (3.98, 4.97)	4.65 (4.06, 5.32)
	Female	2.53 (2.13, 2.95)	2.95 (2.56, 3.36)	3.35 (2.97, 3.77)	3.56 (3.16, 4.02)	3.49 (3.10, 3.87)	4.31 (3.84, 4.79)	4.51 (3.98, 5.12)
	Both	3.26 (2.69, 3.85)	3.60 (3.11, 4.11)	3.88 (3.51, 4.26)	4.01 (3.69, 4.32)	3.77 (3.51, 4.07)	4.38 (4.03, 4.75)	4.58 (4.13, 5.07)
MIR	Male	0.5814	0.5825	0.5851	0.5817	0.5700	0.5615	0.5546
	Female	0.5568	0.5496	0.5472	0.5414	0.5293	0.5248	0.5186
	Both	0.5697	0.5685	0.5695	0.5655	0.5542	0.5469	0.5402

UI, Uncertainty intervals.

**Table 2:** The joinpoint regression model output for the incidence rate trend analysis of lip and oral cavity cancer in Iran between 1990 and 2019 for males, females, and both based on the global burden of disease database.

Segments	Male (2 change points)		Female (3 change points)		Both (3 change points)	
	Time interval	APC (95% CI)	Time interval	APC (95% CI)	Time interval	APC (95% CI)
Trend 1	1990–2004	0.21* (0.08, 0.36)	1990–2002	1.85* (1.68, 2.02)	1990–2002	0.93* (0.76, 1.11)
Trend 2	2004–2011	-1.47* (-2.13, -1.09)	2002–2010	-0.38* (-0.74, -0.10)	2002–2011	-0.85* (-1.23, -0.61)
Trend 3	2011–2019	0.95* (0.65, 1.33)	2010–2015	3.91* (3.32, 4.78)	2011–2015	2.70* (1.92, 3.91)
Trend 4	-	-	2015–2019	0.25 (-0.67, 0.99)	2015–2019	0.26 (-0.89, 0.86)
AAPC	1990–2019	0.007 (-0.05, 0.07)	1990–2019	1.36* (1.29, 1.42)	1990–2019	0.52* (0.44, 0.58)

*Significant at 0.05 level; APC: Annual percent changes; CI: Confidence interval.

**Table 3:** The joinpoint regression model output for the prevalence rate trend analysis of lip and oral cavity cancer in Iran between 1990 and 2019 for male, female and both together based on the global burden of disease database.

Segments	Male (2 join points)		Female (3 join points)		Both (2 join points)	
	Time interval	APC (95% CI)	Time interval	APC (95% CI)	Time interval	APC (95% CI)
Trend 1	1990–2002	1.16* (0.88, 1.48)	1990–2002	2.86* (2.57, 3.15)	1990–2002	1.83* (1.53, 2.17)
Trend 2	2002–2011	-1.20* (-2.14, -0.77)	2002–2010	-0.46 (-1.26, 0.05)	2002–2010	-0.93* (-2.09, -0.37)
Trend 3	2011–2019	1.87* (1.33, 2.67)	2010–2015	4.54 * (3.59, 6.63)	2010–2019	2.38* (1.89, 3.02)
Trend 4	-	-	2015–2019	1.16 * (-0.89, 2.24)	-	-
AAPC	1990–2019	1.23* (0.51, 0.72)	1990–2019	1.98* (1.85, 2.09)	1990–2019	1.23* (1.12, 1.34)

*Significant at 0.05 level; APC: Annual percentage change.

**Table 4:** The joinpoint regression model output for the trend analysis of the mortality-to-incidence (MIR) ratio of lip and oral cavity cancer in Iran between 1990 and 2019 for male, female, and both together based on the global burden of disease database.

Segments	Male (2 join points)		Female (2 join points)		Both (2 join points)	
	Time interval	APC (95% CI)	Time interval	APC (95% CI)	Time interval	APC (95% CI)
Trend 1	1990–2002	-0.68* (-0.78, -0.60)	1990–2002	-1.05* (-1.28, -0.88)	1990–2003	-0.74* (-0.81, -0.67)
Trend 2	2002–2010	0,24* (0.08, 0.51)	2002–2010	0.14 (-0.15, 1.23)	2003–2010	0.28* (0.10, 0.60)
Trend 3	2010–2019	-0.79* (-0.94, -0.67)	2010–2019	-0.79* (-1.13, -0.57)	2010–2019	-0.86* (-0.99, -0.74)
AAPC	1990–2019	-0.46* (-0.49, -0.43)	1990–2019	-0.64* (-0.71, -0.59)	1990–2019	-0.53* (-0.55, -0.50)

*Significant at 0.05 level; APC, Annual percentage change.

**Table 5:** The incidence rate of lip and oral cavity cancer in different age groups in Iran between 1990 and 2019 for male, female, and both from the global burden of disease database.

Segments	Male		Female		Both	
	Time interval	APC (95% CI)	Time interval	APC (95% CI)	Time interval	APC (95% CI)
15–49 years						
Trend 1	1990–1992	2.4 (-1.1, 6.0)	1990–2002	5.8*(5.5, 6.0)	1990–2003	5.3*(5.1, 5.5)
Trend 2	1992–2003	4.9*(4.7, 5.2)	2002–2010	2.4*(1.8, 2.9)	2003–2010	2.1*(1.5, 2.8)
Trend 3	2003–2010	2.0*(1.4, 2.6)	2010–2015	6.3*(5.0, 7.7)	2010–2015	5.2*(3.9,6.5)
Trend 4	2010–2015	3.9* (2.7, 5.0)	2015–2019	1.9*(0.6, 3.2)	2015–2019	2.0*(0.8, 3.3)
Trend 5	2015–2019	2.1*(1.0, 3.3)				
AAPC	1990–2019	3.5*(3.1, 3.9)	1990–2019	4.4* (4.1, 4.7)	1990–2019	-4.0* (3.7, 4.4)
50–69 years						
Trend 1	1990–1999	1.1*(1.0, 1.3)	1990–1999	3.4*(3.2, 3.5)	1990–1999	2.0*(1.8, 2.1)
Trend 2	1999–2002	2.5*(0.5, 4.4)	1999–2002	4.6*(2.7, 6.4)	1999–2002	3.2*(1.4,5.0)
Trend 3	2002–2010	0.7*(0.5, 0.9)	2002–2010	2.9*(2.6, 3.1)	2002–2011	1.8*(1.6,2.0)
Trend 4	2010–2015	5.9*(4.9, 6.9)	2010–2015	6.9*(6.3, 7.5)	2011–2015	6.8*(5.9, 7.8)
Trend 5	2015–2019	4.3*(3.6, 4.9)	2015–2019	4.1*(3.5, 4.7)	2015–2019	4.3*(3.7, 4.8)
AAPC	1990–2019	2.2*(2.0, 2.5)	1990–2019	4.1*(3.8, 4.3)	1990–2019	3.0*(2.8,3.2)
70 + years						
Trend 1	1990–2004	3.6*(3.5, 3.7)	1990–2001	4.7*(4.5, 4.9)	1990–2002	5.3*(5.1, 5.5)
Trend 2	2004–2012	1.5*(1.2, 1.9)	2001–2010	3.6*(3.3, 4.0)	2002–2012	3.2*(3.0, 3.5)
Trend 3	2012–2019	2.8*(2.4, 3.2)	2010–2015	7.2*(6.2, 8.1)	2012–2015	5.8*(3.1, 8.6)
Trend 4	-	-	2015–2019	2.3* (1.3, 3.2)	2015–2019	2.5*(1.7, 3.3)
AAPC	1990–2019	2.8*(2.7, 3.0)	1990–2019	4.4*(4.2, 4.7)	1990–2019	4.3*(3.9, 4.6)

AAPC, Average annual percentage change.

**Table 6:** The years of life lost (YLL), years lived with disability (YLD), and disability adjusted life years (DALY) rates per 100,000 (95% uncertainty intervals (UI)) of lip and oral cavity cancer in Iran for the selected years from the global burden of disease database.

	Sex	1990	1995	2000	2005	2010	2015	2019
YLL	Male	20.99 (16.74, 25.51)	20.38 (17.19, 23.89)	20.08 (17.96, 22.31)	19.75 (18.65, 20.91)	18.22 (17.17, 19.36)	18.40 (17.17, 19.72)	17.74 (15.99, 19.81)
	Female	11.55 (9.73, 13.05)	11.89 (10.48, 13.19)	12.36 (11.56, 13.27)	12.33 (11.59, 13.13)	12.12 (11.36, 12.82)	13.73 (12.85, 14.70)	13.24 (12.20, 14.41)
	Both	16.45 (13.56, 19.05)	16.32 (14.27, 18.46)	16.39 (15.10, 17.64)	16.14 (15.38, 16.91)	15.17 (14.40, 15.90)	16.04 (15.19, 16.98)	15.48 (14.24, 16.87)
YLD	Male	0.50 (0.33, 0.73)	0.52 (0.35, 0.71)	0.53 (0.37, 0.71)	0.53 (0.38, 0.70)	0.49 (0.35, 0.65)	0.52 (0.37, 0.70)	0.53 (0.38, 0.72)
	Female	0.29 (0.20, 0.40)	0.33 (0.23, 0.45)	0.37 (0.26, 0.49)	0.38 (0.27, 0.51)	0.38 (0.27, 0.50)	0.46 (0.32, 0.60)	0.47 (0.33, 0.61)
	Both	0.40 (0.28, 0.56)	0.43 (0.30, 0.58)	0.45 (0.32, 0.60)	0.46 (0.33, 0.61)	0.43 (0.31, 0.57)	0.49 (0.35, 0.64)	0.50 (0.36, 0.66)
DALY	Male	21.50 (17.12, 26.11)	20.90 (17.62, 24.48)	20.61 (18.47, 22.86)	20.28 (19.16, 21.48)	18.71 (17.67, 19.88)	18.93 (17.67, 20.30)	18.27 (16.48, 20.42)
	Female	11.85 (9.97, 13.41)	12.22 (10.79, 13.50)	12.73 (11.90, 13.67)	12.71 (11.95, 13.53)	12.49 (11.73, 13.21)	14.18 (13.29, 15.21)	13.71 (12.63, 14.91)
	Both	16.85 (13.89, 19.53)	16.75 (14.64, 18.89)	16.84 (15.49, 18.15)	16.60 (15.81, 17.38)	15.60 (14.81, 16.30)	16.53 (15.65, 17.51)	15.98 (14.70, 17.44)

**Table 7:** The joinpoint regression model output for the trend analysis of the years of life lost (YLL), years lived with disability (YLD), and disability adjusted life years (DALY) of lip and oral cavity cancer in Iran between 1990 and 2019 for male, female, and both based on the global burden of disease database.

Index	Segments	Male		Female		Both	
		Time interval	APC (95% CI)	Time interval	APC (95% CI)	Time interval	APC (95% CI)
YLL	Trend 1	1990–1999	-0.45* (-0.60, -0.36)	1990–2002	0.73* (0.65, 0.80)	1990–2003	0.03 (-0.03, 0.10)
	Trend 2	1999–2002	0.17 (-0.49, 0.35)	2002–2008	-0.75* (-1.12, -0.51)	2003–2011	-1.12* (-1.28, -0.98)
	Trend 3	2002–2005	-0.63* (-1.59, -0.05)	2008–2011	0.73 (-0.29, 1.82)	2011–2015	1.62* (1.29, 1.92)
	Trend 4	2005–2011	-1.67* (-1.83, -1.51)	2011–2015	2.93* (2.66, 3.43)	2015–2019	-0.92* (-1.25, -0.61)
	Trend 5	2011–2015	0.57* (0.35, 0.80)	2015–2019	-1.04* (-1.32, -0.78)	-	-
	Trend 6	2015–2019	-0.87* (-1.12, -0.62)	-	-	-	-
	AAPC	1999–2019	-0.58* (-0.60, -0.56)	1999–2019	0.48* (0.44, 0.50)	1999–2019	-0.20* (-0.23, -0.17)
YLD	Trend 1	1990–2003	0.57* (0.36, 0.80)	1990–2002	2.23* (2.02, 2.45)	1990–2002	1.26* (1.02, 1.51)
	Trend 2	2003–2011	-1.36* (-2.32, -0.94)	2002–2010	-0.43* (-0.94, -0.07)	2002–2011	-0.88* (-1.47, -0.24)
	Trend 3	2011–2019	1.33* (0.89, 1.93)	2010–2015	4.14* (3.41, 5.53)	2011–2015	3.12 (-0.72, 4.61)
	Trend 4	-	-	2015–2019	0.65 (--0.65, 1.53)	2015–2019	0.65 (-1.03, 1.79)
	AAPC	1999–2019	0.24* (0.15, 0.33)	1999–2019	1.60* (1.50, 1.67)	1999–2019	0.76* (0.64, 0.84)
DALY	Trend 1	1990–1999	-0.43* (-0.57, -0.35)	1990–2002	0.74* (0.66, 0.83)	1990–2003	0.06 (-0.006, 0.14)
	Trend 2	1999–2002	0.20 (-0.45, 0.37)	2002–2010	-0.56* (-0.71, -0.43)	2003–2011	-1.12* (-1.28, -0.98)
	Trend 3	2002–2005	-0.63* (-1.54, -0.04)	2010–2015	2,86* (2.68, 3.05)	2011–2015	1.68* (1.35, 1.99)
	Trend 4	2005–2011	-1.67* (-1.82, -1.54)	2015–2019	-0.94* (-1.21, -0.67)	2015–2019	-0.88* (-1.21, -0.56)
	Trend 5	2011–2015	0.61* (0.39, 0.84)	-	-	-	-
	Trend 6	2015–2019	-0.83* (-1.08, -0.59)	-	-	-	-
	AAPC	1999–2019	-0.56* (-0.58, -0.54)	1999–2019	0.51* (0.48, 0.54)	1999–2019	-0.17* (-0.21, -0.14)

*Significant at 0.05 level; AAPC, Average annual percentage change.

## Discussion

In this study, we aimed to epidemiologically describe the incidence, morbidity, mortality, and burden trends of LOCCs in Iran from 1990 to 2019 according to GBD. In general, during these 30 years, there has been a significant increase in the incidence of LOCC in Iran, which has mostly been related to the much higher rate of increase in the incidence of the disease in women. This increase in the incidence of LOCC in Iranian women took place between 1990 and 2002 and between 2010 and 2015. However, there were increases in the LOCC incidence in men from 1990 to 2004 and 2011 to 2019. If we consider age groups, LOCC has increased in all age groups, although the highest increase in both sexes was in the middle-aged group. The growth of LOCC incidence in middle-aged men has been more than in middle-aged women, and the incidence of the disease has been growing rapidly in middle-aged men from 2011 to 2019.

The two most established global online cancer databases are Global Cancer Observatory (GLOBOCAN 2020) and GBD 2019. Comparing the information of the above two sources with a special focus on oral cancer, Iran was in the group of countries where only minor differences could be observed between the two abovementioned databases, which can be a sign of accurate recording of cancer cases in the country and the correct governance system in this field^23^.

Our finding that the incidence of LOCC increased during the studied years was consistent with the global increase in these cancers^24^. Sun et al. showed that global ASIR of oral cancer increased in females but decreased in males^24^. In a recent systematic review, the pooled ASIR of oral cavity cancer for men and women was estimated to be 1.96 and 1.46, respectively. Moreover, based on Trim-and-fill methods, overall ASIR corrected in women was estimated at 1.36. It was concluded that the incidence of oral cavity cancer in Iran has been lower than the global average; however, due to the increasing age of the population and exposure to risk factors, an increasing trend can be anticipated in the future^6^.

In this investigation, middle-aged populations of both genders experienced higher rates of incidence compared to other age groups. In a previous multicenter study from 2005 to 2014, the mean age of the patients was about 58 years^25^. Moreover, according to a previous systematic review on oral cancer in Iran in 2015, the mean age of patients in 25 studies was 54 years and the male/female ratio of patients was 1.91. In addition, Iran seemed to have a similar epidemiological pattern of oral cancer to other countries in the world^26^.

Despite the increased incidence rate of LOCC in the Iranian population, the mortality-to-incidence ratio has been declining in each of the two sexes and in both together throughout the study period except from 2002 to 2010. A decrease in the MIR as a multifactorial indicator may show an improvement in the quality of diagnosis and treatment of cancers and the patient’s care in the period under review^4^. The overall prognosis of oral cancer is poor; although, early diagnosis in the early stages (I and II) improved survival rates by about 80%^27^. It seems that improvement in diagnosis and treatment methods has positive effects on the survival of patients with LOCC in the Iranian population.

In the present study, the trend of the years of life lost (YLL) showed the most heterogeneity and join points. A significant decrease in men, a significant increase in women, and a low significant decrease in both sexes together can be observed in the YLL trend. The increase in YLL due to LOCC in women is consistent with the increase in the incidence of the disease in them. In addition, the model of changes in the DALY index is also more similar to YLL than to YLD, which could be related to high mortality of oral cancer rather than disabilities caused in surviving patients. According to a previous investigation, premature death in men had the most important role (62%) in the lost productivity caused by oral cancer in Iran in 2014^8^. We observed that DALY of the total patients shows a low significant decreasing trend. The trend of DALY in men shows a general significant decrease, despite a generally significant increasing trend of DALY in the affected women. The increasing trend of DALY in female patients is consistent with the increase in the incidence of LOCC in this gender. In general, changes in the DALY of LOCC can have many variations in different geographical regions. O’Sullivan et al. showed significant geographic variations in age-standardized DALYs of lip and oral cavity, nasopharynx, and other pharynx cancers (LOCP) in Europe from 1990 to 2019^18^. During the 30 years of this observation in Europe, the ASMR and DALY rates decreased by 16% and 21%, respectively^18^. The above results show that it is possible to reduce the mortality and disability caused by LOCC; thus, planning to minimize exposure to risk factors and improve screening, diagnosis, and treatment methods for LOCC in Iran should be seriously followed.

Worldwide, the prevalence of oral carcinoma is noticeably elevated among females rather than males; especially in developing countries^28,29^. A study in 2020 showed that the incidence and the ASIR of oral cancer have significantly increased all over the world, while age-standardized DALYs have remained unchanged or decreased^30^. DALY not changing or remaining stable may be related to the fact that the general trend of the quality of care regarding oral cancer has improved worldwide^15^. However, the above data have been varied in different regions of the world and different countries^30^. Pakistan and India have the highest increase of ASIR, ASMR, and ASR-DALY in oral cancer which can be due to population growth, dietary habits, oral hygiene, and citizens’ behavior. In general, paying more attention to oral hygiene and the habits and behavior of citizens by governments can help reduce the incidence of oral cancer^30–32^.

The sex-related increase in LOCC incidence in Iranian women can probably be assumed due to changes in their risk factor trends. However, smoking is still much less common among Iranian women than men. According to a national update from the STEPS survey 2021^33^, the all-ages prevalence (95% CI) of current tobacco smoking in Iran was 25.88% (25.03–26.75) among men, 4.44% (4.09–4.82) among women, and 14.01% (13.56–14.48) overall. The all-ages prevalence of current cigarette smoking was 19.95% (19.17–20.75) among men, 0.77% (0.62–0.95) among women, and 9.33% (8.95–9.72) overall^33^. As a recent systematic analysis of GBD 2019 data has shown, smoking and alcohol play the most enormous role in LOCC deaths in male individuals around the world while chewing tobacco has played the biggest role in affected female individuals among all risk-attributable deaths caused by LOCC^34^. In Iran, some habits and behaviors can be considered risk factors for oral cancer. Traditional water pipe (hookah) is smoked in many cafes and restaurants in Iran. The all-ages prevalence of current hookah smoking was 3.64% (3.33, 3.98) among women, 5.56% (5.12–6.03) among men, and 4.5% (4.23–4.78) overall^33^. Another problem in Iran is smokeless tobacco use known as Nass (Snus)^28,35^. It is also used in Central Asia, Afghanistan, and Pakistan. Family history of its usage and unemployment can be associated with Nass use^36^. Additionally, other various types of smokeless tobacco consumed in Iran have been Pan, Gutka, Naswar, Biti, and Supari. Most consumers people live in Sistan-Baluchestan and Golestan Provinces^37^. The results of a systematic review showed a strong link between the usage of different forms of smokeless tobacco and oral cancer^5^. In a case-control study, the use of tobacco products such as Nass, pipe, and hookah could increase the odds of getting LOCC by 4 times^38^. Women were also part of the smokeless tobacco consumers who often hid their consumption which can alarm their health^37^. Another public health concern in Iran is drug abuse. Opium (*Lachryma papaveris*) and its derivatives are the most commonly used drugs in Iran. The World Health Organization (WHO) estimated that about 2 million Iranians abuse opium, which is three times higher than the global average^39,40^. Opium use has been associated with an increased risk of LOCC showing a dose-response relation with the amount of daily use^38^.

Another condition that has been suggested as an independent risk factor for the development of oral cancer is periodontal disease^41–43^, followed by poor oral hygiene and tooth loss^43^. Good oral hygiene habits such as daily brushing with toothpaste and annual dental check-ups which result in healthy gingiva and a minimal tooth loss can significantly reduce the risk of oral cancer^44^. In different studies, the oral health status of the Iranian adult population was poor and undesirable^45–47^, and between 1990 and 2010 in Iran, an increase occurred in DALYs of periodontal disease at all ages^48^. As the results of a systematic review revealed, 48.7% of elderly individuals in Iran were completely edentulous^49^. In the Golestan cohort study on people aged 40 to 75 years in northeastern Iran, participants with poorer oral health and more tooth loss had an increased risk of mortality from cancer^50^. Moreover, among people who had poor oral health, the risk of developing upper gastrointestinal cancers was higher in the abovementioned study^51^. The association between tooth loss and increased mortality, especially in the case of cancers, can be through changes in the oral microbiome^50^. Disruptions in the equilibrium of the oral microbiome by dietary habits and inadequate oral hygiene can result in different oral diseases like periodontitis, dental caries, and even oral cancer^52^. Other risk factors of oral cancer include geographic variation, genetic predisposition, diets, immune status, oncogenic viruses, radiation, and environmental factors^28,53–55^. Diets rich in fresh fruits and vegetables, micronutrients and vitamins A, C, and E may lead to protection against oral cancer^56,57^.

There has been more awareness about the risk factor of oral cancer regarding tobacco use and cigarettes smoking than other risk factors in the people of Tehran (the capital of Iran), and knowledge of initial signs has been deficient^58^. Educating people is the most feasible way to make positive changes by giving information about habitual risk factors and initial signs and symptoms which can improve timely diagnosis^58^. People who have a lower socio-economic status, illiteracy, diabetes, smoking, or drug addiction have the greatest need for education to improve oral hygiene/health care^45,46,59^. Finally, increasing oral health literacy, growing public awareness regarding the risks of consumption of various tobacco products, cigarettes, and opioids, especially for women who have been exposed to the abovementioned risk factors, changing behavioral habits, promoting socioeconomic status, and enhancing people’s access to a healthy and nutritious diet and health care/insurance can improve oral health and reduce oral cancer incidence in Iran^38,45,47^.

The data obtained from the GBD study fills the gap in our knowledge about diseases burden with a globally acceptable quality and create a general comprehension of the amount of deaths/disabilities caused to humans due to various health problems. However, the inevitable limitations of the above data should be kept in mind^60^. First, the accuracy and robustness of the estimates in GBD are largely dependent on the quantity and quality of the data used in the modeling. For example, data on the collection of lip and oral cavity cancers, not each one separately, are simultaneously collected/reviewed/estimated in the GBD. The abovementioned data mainly described the overall trends of LOCC, regardless of the lesion site and its histopathological type^30,60^. Second, in GBD data, risk factors are not assessed. For example, risk factors of lip and oral cancer have not been analyzed, so their exact role along with the geographic distribution patterns of the disease cannot be investigated^30,61^. Third, some variations observed in mortality and DALY could be partially related to the bias in detecting the relevant statistics. This detection bias could be linked with the modifications in disease diagnosis and screening protocols in different countries or the disappointing population coverage in some low-income regions; although many adjusted methods have been conducted in GBD studies to reduce such bias^60^.

We suggest for future studies that “Quality of Care” indicators be used to investigate which age/sex groups have received better diagnostic/treatment/care services and which have been deprived. And whether the deprived group was associated with increased MIR/YLL/YLD/DALY indices. It is also suggested to obtain population indicators in Iran such as the Sociodemographic Index (SDI) or Human Development Index (HDI) and measure their association with mortality rate and the abovementioned morbidity indicators.

## Conclusions

According to GBD, the incidence rate of LOCC has been increasing in the Iranian population; this ascendance is greater in female patients and the 50-69-year-old age group. Paying attention to these groups for early detection of LOCC improves their survival. Moreover, assessment of the changes in risk factors in the abovementioned groups may be helpful for disease prevention. Generally, YLL and DALY of the total patients showed a significant decreasing trend. Moreover, in this thirty-year studied period, except for the time range of 2002–2010, MIR has been declining which can show an improvement in screening, diagnosis, treatment, and patient care of LOCC in Iran.

## Electronic supplementary material

Below is the link to the electronic supplementary material.

Supplementary Material 1

Supplementary Material 2

Supplementary Material 3

Supplementary Material 4

## Data Availability

The data that support the findings of this study are available in “Global Burden of Disease Study” (GBD) database at http://ghdx.healthdata.org/gbd-results-tool. These data were derived from the following resources available in the public domain: - “Global Burden of Disease Study” (GBD) database, http://ghdx.healthdata.org/gbd-results-tool.

## Abbreviations

LOCC: Lips and oral cavity cancers
ICD: International statistical classification of diseases and related health problems
SCC: Squamous cell carcinoma
GBD: Global burden of disease
IHME: Institute for health metrics and evaluation
ASIRs: Age-standardized incidence rates
ASMR: Age-standardized mortality rate
YLL: Years of life lost
YLD: Years lived with disability
DALY: Disability adjusted life years
MIR: Mortality-to-incidence ratio
CODEm: Cause of death ensemble model
UI: Uncertainty intervals
APC: Annual percent change
AAPC: Average annual percent change
CI: Confidence interval
GLOBOCAN: Global cancer observatory
ASR: Age-standardized rate
STEPS: STEPwise approach to non-communicable disease risk factor surveillance
WHO: World Health Organization
CPITN: The community periodontal index of treatment needs
