# Supplementary material for: Lip and oral cavity cancer in Iran from 1990 to 2019 based on the global burden of disease study
Source: Sci Rep. 2025 Mar 3;15:7389. doi: 10.1038/s41598-025-92090-w (PMC11876645; doi:10.1038/s41598-025-92090-w)

|  |  |  |  |  |  |  |  |
| --- | --- | --- | --- | --- | --- | --- | --- |
|  |  |  |  |  |  |  |  |

**Appendix Image 1.** The output of the analysis performed by the Joinpoint software on the incidence data of lip and oral cavity cancer in Iran from 1990 to 2019.
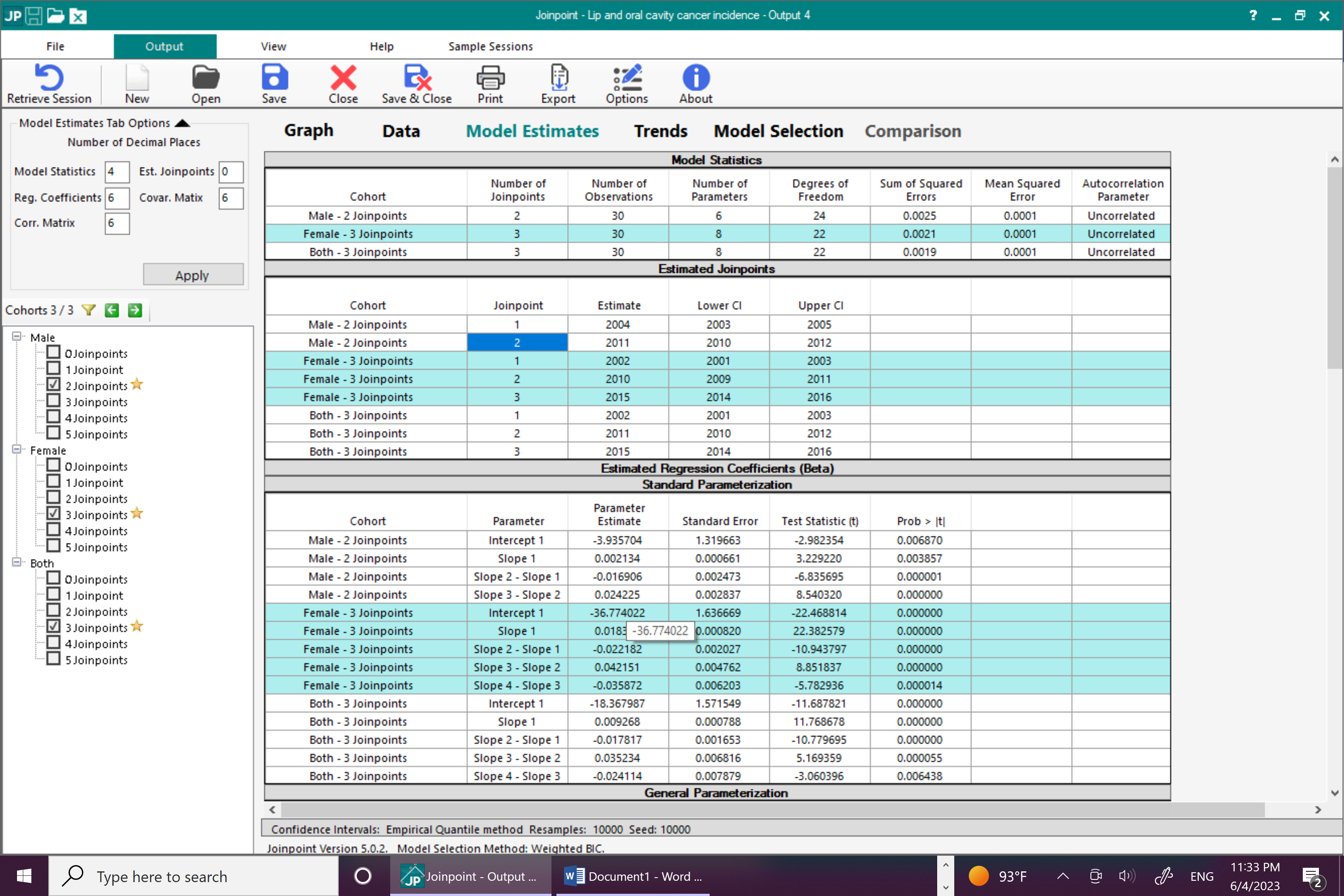


s
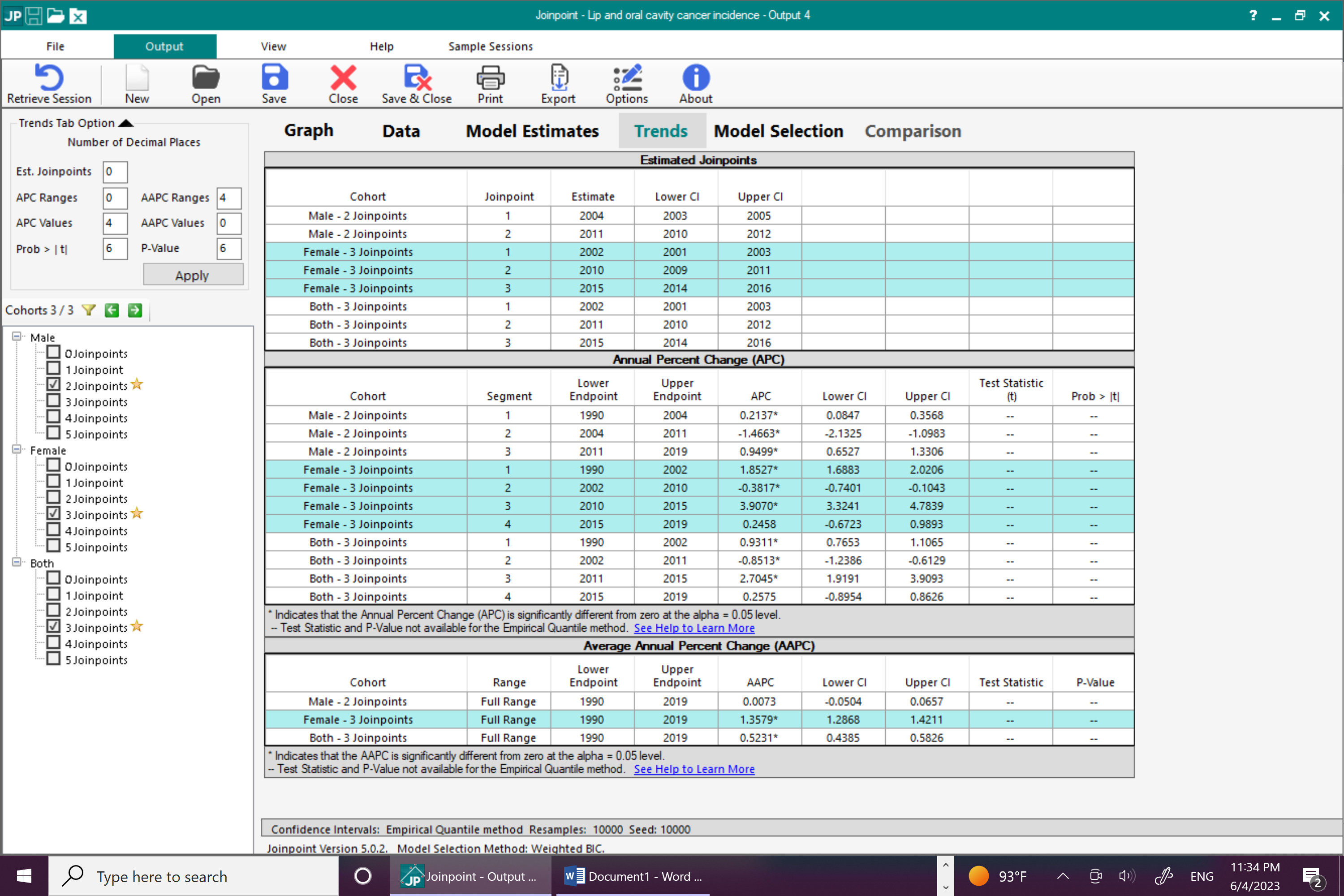

Supplement: Supplementary file 1 — Supplementary Material 1 [file 41598_2025_92090_MOESM1_ESM.docx]
