# Supplementary material for: Lip and oral cavity cancer in Iran from 1990 to 2019 based on the global burden of disease study
Source: Sci Rep. 2025 Mar 3;15:7389. doi: 10.1038/s41598-025-92090-w (PMC11876645; doi:10.1038/s41598-025-92090-w)

**Appendix Image 4.** The output of the analysis performed by the Joinpoint software on **A.** the years lived with disability (YLD), **B.** years of life lost (YLL), and **C.** disability adjusted life years (DALY) data of lip and oral cavity cancer in Iran from 1990 to 2019.


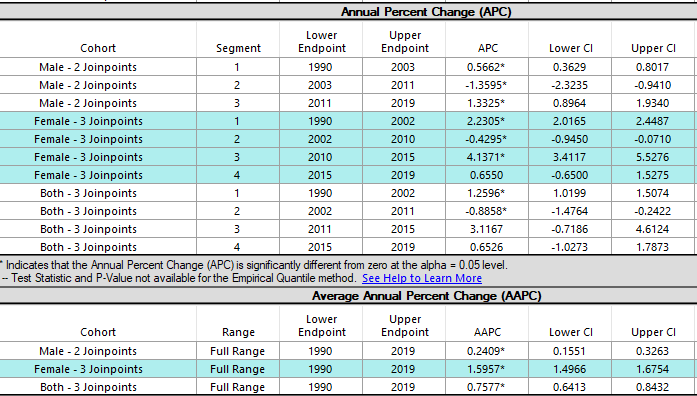


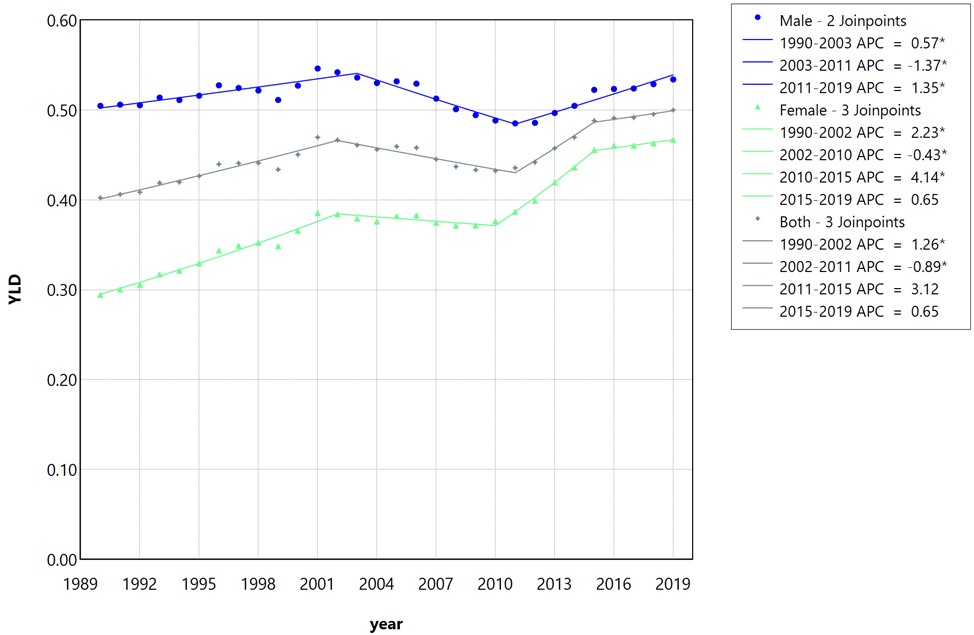


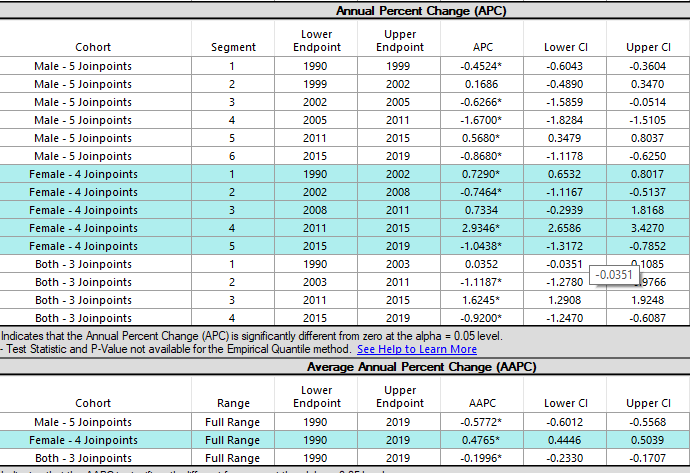


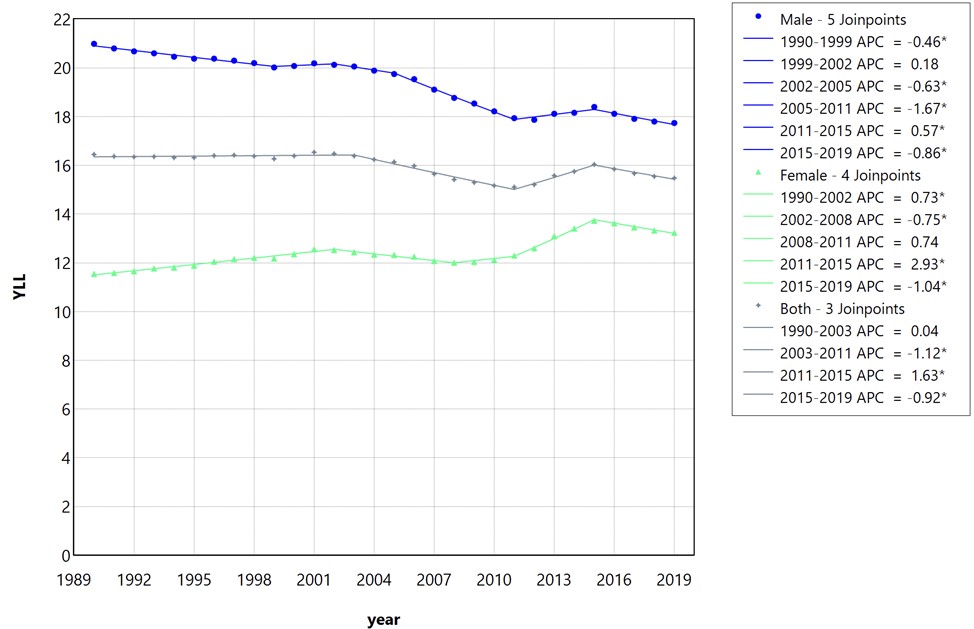


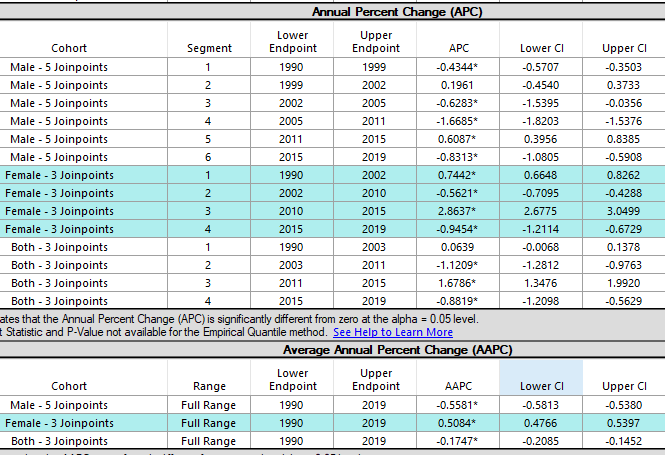


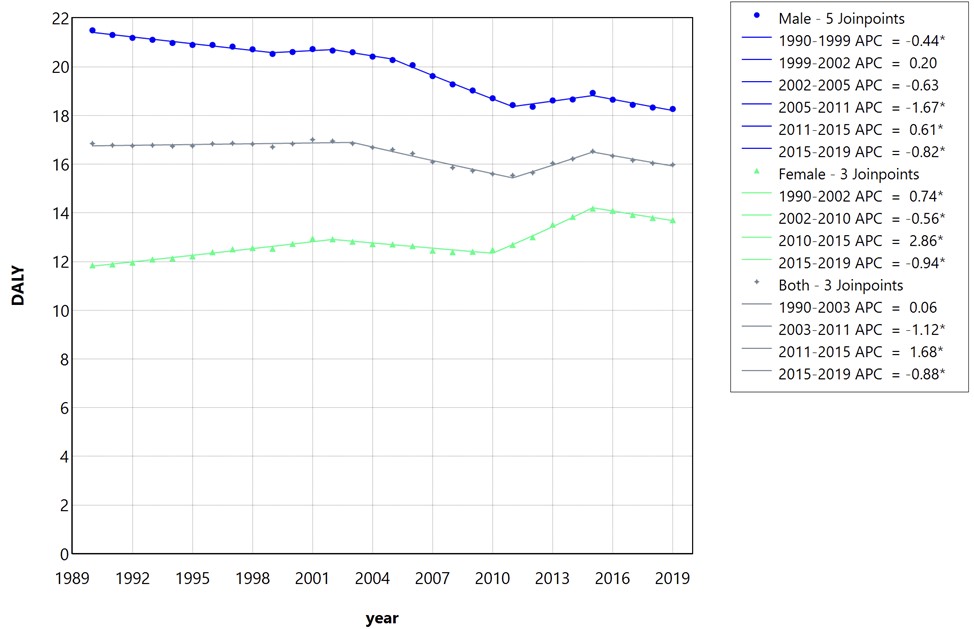

Supplement: Supplementary file 4 — Supplementary Material 4 [file 41598_2025_92090_MOESM4_ESM.docx]
